# *Rickettsia slovaca* in *Dermacentor marginatus* ticks, Germany

**DOI:** 10.3201/eid1512.090843

**Published:** 2009-12

**Authors:** Silvia Pluta, Friedemann Tewald, Kathrin Hartelt, Rainer Oehme, Peter Kimmig, Ute Mackenstedt

**Affiliations:** University of Hohenheim, Stuttgart, Germany (S. Pluta, P. Kimmig, U. Mackenstedt); Baden-Wuerttemberg State Health Office, Stuttgart (S. Pluta, K. Hartelt, R. Oehme); Labor Enders and Partner, Stuttgart (F. Tewald)

**Keywords:** Rickettsia slovaca, Dermacentor marginatus, Rickettsia, tick-borne lymphadenopathy, Germany, letter

**To the Editor:** *Dermacentor* spp. ticks are found in many countries in Europe, and are vectors for several pathogens, including *Francisella tularensis*, *Coxiella burnetii*, *Rickettsia* spp., and *Babesia canis* (*1*,*2*). Because *Dermacentor marginatus* ticks require warm dry habitats, these ticks are found in only a few areas in southern Germany, mainly in the Rhine and Main valleys (*3*). However, these ticks may spread northwards because of increasing temperatures. In contrast, *D*. *reticulatus* ticks are present throughout Germany.

Fourteen *Rickettsia* spp. are currently identified as human pathogens. The severity of human diseases differs among these species, ranging from mild to lethal illness (*4*). On the basis of serologic and genotypical characteristics, rickettsiae are divided into typhus and spotted fever groups. Within each group, antigenic differences are small, resulting in cross-reactivity that complicates differentiation of *Rickettsia* spp. by serologic methods. Therefore, PCR of eschar biopsy samples is a useful tool for diagnosis of rickettsial diseases (*5*).

Little information exists regarding the prevalence of *Rickettsia* spp. in *D*. *marginatus* and *D*. *reticulatus* ticks in Germany. *R*. *raoultii* was recently detected in 23% of *D*. *reticulatus* ticks (*6*). In 1977, Rehacek et al. identified *R*. *slovaca* in 12% of *Dermacentor* spp. ticks from southern Baden-Wuerttemberg (*7*). Since then, this pathogen has not been detected in Germany. *R*. *slovaca*, a member of the spotted fever group, causes tick-borne lymphadenopathy, a relatively mild rickettsiosis (*8*). We report detection of *R*. *slovaca* in 5 of 666 *Dermacentor* spp. ticks from southern Germany. Moreover, we identified a case of tick-borne lymphadenopathy from Rhineland-Palatinate.

We collected 666 adult *Dermacentor* spp. ticks by blanket-dragging; 26 were collected along the Main River near Aschaffenburg (Bavaria), and 640 from the Rhine Valley near Lörrach (Baden-Wuerttemberg). Ticks were homogenized, and the DNA was isolated by using the Maxwell 16 Instrument (Promega, Madison, WI, USA). For detection of rickettsiae, a TaqMan real-time PCR with the LightCycler system (Roche Diagnostics, Mannheim, Germany) was performed according to Wölfel et al. (*9*). A primer pair amplified a 70-bp fragment of the citrate synthase (*gltA*) gene. All positive samples were also tested with a PCR specific for the outer membrane protein A (*rOmpA*) gene (*10*). This amplification yielded an *rOmpA* fragment of 532 bp. Amplified products were analyzed by agarose gel electrophoresis.

For identification of *Rickettsia* spp., amplification products of the *rOmpA* PCR were sequenced by using fluorescence-labeled dideoxynucleotide technology (Applied Biosystems, Darmstadt, Germany). Separation of sequenced fragments and data collection were performed by using an ABI PRISM 310 Genetic Analyzer (Applied Biosystems). All obtained sequences were analyzed and compared by using BLAST (www.ncbi.nlm.nih.gov/BLAST).

*Rickettsia* spp. was detected by both PCR methods in 31% of 666 *Dermacentor* spp. ticks examined. Sequencing of part of the *rOmpA* gene showed that sequences of 5 samples (0.75%) from Aschaffenburg were *R*. *slovaca*, showing 100% similarity with a sequence deposited in GenBank (accession no. U43808). Only *D*. *marginatus* ticks from Aschaffenburg were infected with *R*. *slovaca*, and 202 ticks from Lörrach were infected with *R*. *raoultii*.

We also identified a case of *R*. *slovaca* infection in southern Rhineland-Palatinate. The patient reported a tick bite; the tick was identified as *Dermacentor* spp. Fever, lymphadenopathy of submandibular lymph nodes, and exanthema at the site of the tick bite developed 7 days later. Serologic examinations by using an immunofluorescent test (Focus Diagnostics, Cypress, CA, USA) showed antibody titers of 64 for immunoglobulin (Ig) M and 1,024 for IgG against rickettsiae of the spotted fever group. These results indicated an acute rickettsial infection. Because of strong cross-reactivity among all species in the spotted fever group, we cannot differentiate between antibodies against *R*. *slovaca* and other species in this group.

However, another immunofluorescent test for typhus group rickettsiae showed negative results, confirming that the patient was infected with spotted fever group rickettsiae. Results of PCRs specific for *gltA* and *rOmpA* of the patient’s tick were positive. We identified *R*. *slovaca* by sequencing the *rOmpA* gene. The sequence obtained showed 100% similarity with sequences in ticks from Aschaffenburg. Clinical symptoms, serologic results, and detection of *R*. *slovaca* in the tick from the patient strongly indicate that the patient had tick-borne lymphadenopathy caused by *R*. *slovaca*.

The high prevalence of *R*. *raoultii* in *Dermacentor* spp. ticks is of concern because this species can also cause tick-borne lymphadenopathy, although *R*. *raoultii* is less pathogenic than *R*. *slovaca* (*8*). Tick-borne lymphadenopathy should be considered in the differential diagnosis of tick-borne diseases. The extent of the distribution of *R*. *slovaca* and *R*. *raoultii* in Germany remains to be elucidated.
